# Inhibitory and modulatory inputs to the vocal central pattern generator of a teleost fish

**DOI:** 10.1002/cne.24411

**Published:** 2018-02-28

**Authors:** Elisabeth Rosner, Kevin N. Rohmann, Andrew H. Bass, Boris P. Chagnaud

**Affiliations:** ^1^ Department Biologie II Ludwig‐Maximilians‐University Munich Planegg‐Martinsried 82152 Germany; ^2^ Graduate School of Systemic Neurosciences Munich Planegg‐Martinsried 82152 Germany; ^3^ Department of Neurobiology and Behavior W239/233 Mudd Hall Cornell University Ithaca New York 14853

**Keywords:** dopamine, GABA, gap junction, glycine, inhibition, neuromodulation, serotonin, teleost fish, vocal pattern generation, RRID:AB_2079751, RRID:AB_94632, RRID:AB_94632, RRID:AB_10013381, RRID:AB_2560949, RRID:AB_10718516, RRID:AB_572268, RRID:AB_2340846, RRID:AB_142672, RRID:AB_141708, RRID:AB_2337244

## Abstract

Vocalization is a behavioral feature that is shared among multiple vertebrate lineages, including fish. The temporal patterning of vocal communication signals is set, in part, by central pattern generators (CPGs). Toadfishes are well‐established models for CPG coding of vocalization at the hindbrain level. The vocal CPG comprises three topographically separate nuclei: pre‐pacemaker, pacemaker, motor. While the connectivity between these nuclei is well understood, their neurochemical profile remains largely unexplored. The highly vocal Gulf toadfish, *Opsanus beta*, has been the subject of previous behavioral, neuroanatomical and neurophysiological studies. Combining transneuronal neurobiotin‐labeling with immunohistochemistry, we map the distribution of inhibitory neurotransmitters and neuromodulators along with gap junctions in the vocal CPG of this species. Dense GABAergic and glycinergic label is found throughout the CPG, with labeled somata immediately adjacent to or within CPG nuclei, including a distinct subset of pacemaker neurons co‐labeled with neurobiotin and glycine. Neurobiotin‐labeled motor and pacemaker neurons are densely co‐labeled with the gap junction protein connexin 35/36, supporting the hypothesis that transneuronal neurobiotin‐labeling occurs, at least in part, via gap junction coupling. Serotonergic and catecholaminergic label is also robust within the entire vocal CPG, with additional cholinergic label in pacemaker and prepacemaker nuclei. Likely sources of these putative modulatory inputs are neurons within or immediately adjacent to vocal CPG neurons. Together with prior neurophysiological investigations, the results reveal potential mechanisms for generating multiple classes of social context‐dependent vocalizations with widely divergent temporal and spectral properties.

## 1 INTRODUCTION

Vocal behavior frequently plays a central role in intra and interspecific interactions among vertebrates. Vocalizations often exhibit high temporal precision resulting in the transmission of highly stereotyped signals between sender and receiver (Bradbury & Vehrencamp, 2011). The neuronal circuitry that generates these signals includes forebrain and midbrain regions and a single or multiple hindbrain vocal central pattern generators (CPGs, e.g., Bass, 2014; Chakraborty & Jarvis, 2015).

While forebrain and midbrain regions underlying vocal behavior have received considerable attention in vertebrate lineages as diverse as birds and primates, hindbrain vocal circuits remain largely unexplored despite their undisputed role in vocal patterning (e.g., Hage & Jürgens, 2006; Jürgens, 2002; Levelt, 1989; Suthers & Goller, 1997). Evidence from electrophysiological studies in bats and monkeys, for instance, show the contribution of hindbrain neurons to the determination of the amplitude, duration, and frequency of vocal signals (Jürgens & Hage, 2007; Rübsamen & Betz, 1986; Schuller & Rübsamen, 1990; Suthers & Goller, 1997; Vicario, 1991). The most comprehensive investigations of vocal CPGs, however, originate from studies of amphibians (Kelley et al., 2017; Schmidt, 1992; Yamaguchi, Kaczmarek, & Kelley, 2000; Zornik & Yamaguchi, 2012) and fish (Bass, 2014; Bass & Baker, 1990; Bass, Chagnaud, & Feng, 2015).

**Figure 1:**
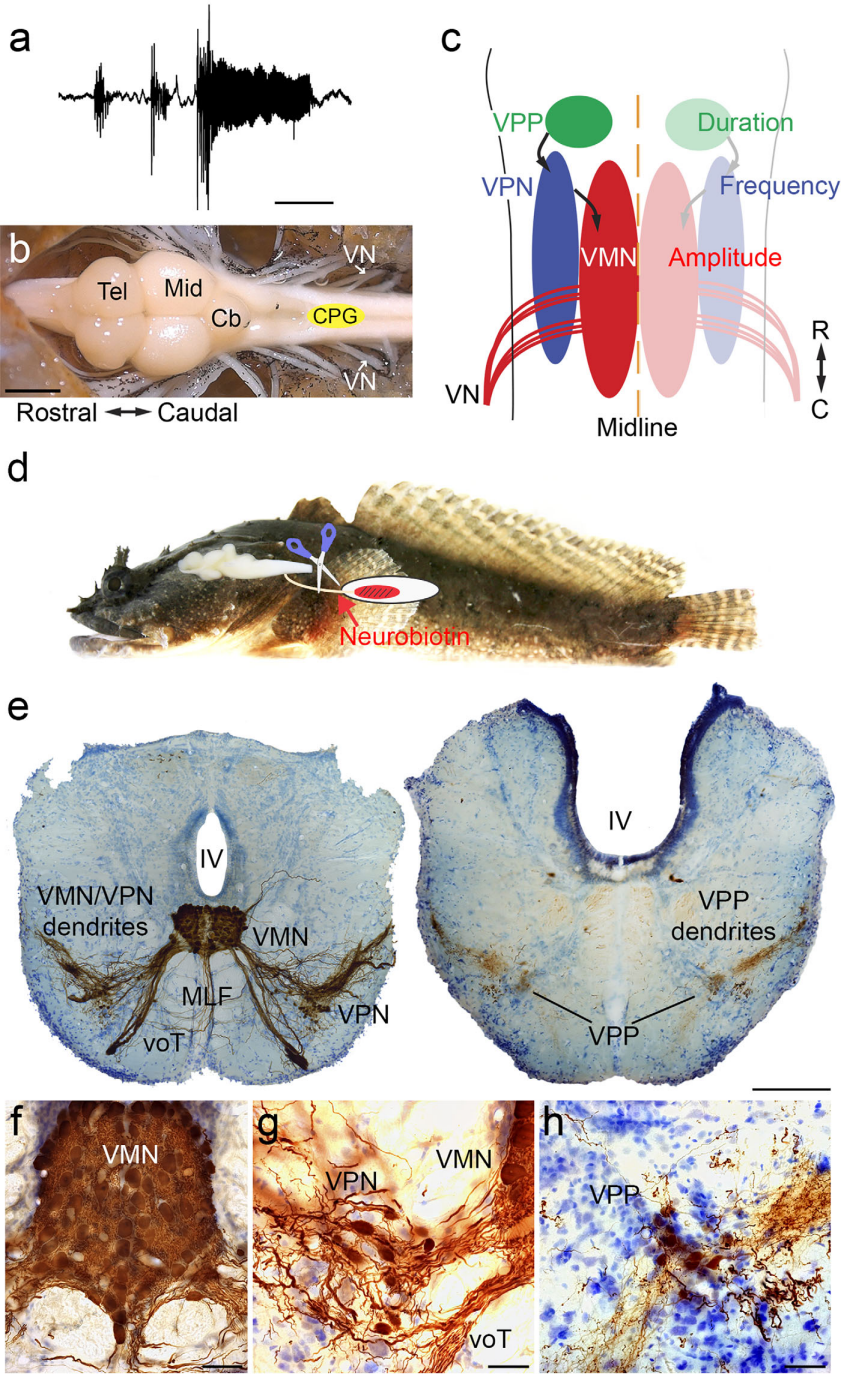
Organization of the Gulf toadfish (*Opsanus beta*) vocal CPG. (a) Hydrophone recording of a boatwhistle, a toadfish advertisement call (L. Remage‐Healy & A. H. Bass, unpublished obs.). (b) Photograph of a dorsal view of a toadfish brain showing the relative location of the vocal central pattern generator (CPG, yellow ellipse). Rostral‐caudal axis is indicated. (c) Schematic (dorsal view) of the toadfish vocal CPG. The left side displays the individual nuclei (vocal pre‐pacemaker nucleus, VPP; vocal pacemaker nucleus, VPN; vocal motor nucleus, VMN). The right side indicates the vocal property that is encoded by each nucleus (color coded). Rostral (R)‐caudal (C) axis is indicated. (d) Side view of Gulf toadfish (modified from Bass and Rice, 2010) and brain with schematic drawing illustrating neurobiotin labeling at the cut end of the vocal nerve that innervates the vocal muscle (red) attached to the wall of the swim bladder (white). (e) Photographs of transverse hindbrain sections with transneuronal neurobiotin labeling of vocal CPG and cresyl violet counterstain at the level of the VMN/VPN circuit (left) and of the VPP (right). (f‐h) Higher magnification photographs of VMN (f), VPN (g), and VPP (h). Scale bars represent 100 ms (a), 2 mm (b), 200 µm (e), 50 µm (f) and 20 µm (g, h). Abbreviations: IV, fourth ventricle; Cb, cerebellum; Mid, midbrain; MLF, medial longitudinal fasciculus; Tel, telencephalon; VN, vocal nerve; voT, vocal tract

Among vocal species of fish, toadfishes (order Batrachoidiformes, family Batrachoididae) include species commonly known as toadfish and midshipman fish (Greenfield, Winterbottom, & Collette, 2008) that have provided tractable models for neurophysiological investigations of vocal CPGs (Ladich, Collin, Moller, & Kapoor, 2006). These fish produce several types of social context‐dependent vocalizations (e.g., Figure 1a) by repetitively contracting a single pair of “superfast” vocal muscles attached to the walls of the swim bladder at frequencies of about 100–250 Hz depending on the ambient water temperature (Bass & Baker, 1991; Brantley & Bass, 1994; Cohen & Winn, 1967; Rome, 2006; Skoglund, 1961). These contractions are elicited by the activity of a hindbrain vocal motor nucleus (VMN) that innervates the vocal muscles via occipital motor nerve roots. These roots form a single vocal nerve (VN, Figure 1b, c) considered homologous to the hypoglossal nerve roots of other vertebrates (Bass, Gilland, & Baker, 2008). Transneuronal tracing via labeling of the cut end of one VN with neurobiotin or biocytin (Figure 1d, e) together with single cell electrophysiological recordings demonstrate three topographically separate vocal CPG nuclei that code for distinct call parameters: VMN for amplitude modulation, vocal pacemaker nucleus (VPN) for pulse repetition rate (corresponds to fundamental frequency) and vocal pre‐pacemaker nucleus (VPP) for duration (Figure 1f–h; Bass & Baker, 1990; Bass, Marchaterre, & Baker, 1994; Chagnaud, Baker, & Bass, 2011; Chagnaud, Zee, Baker, & Bass, 2012).

While the organization of vocal CPGs has been investigated at network and single neuron levels for toadfishes and the African clawed frog *Xenopus laevis* (e.g., Bass & Baker, 1990; Chagnaud et al., 2011; Chagnaud et al., 2012; Kelley et al., 2017; Yamaguchi et al., 2000; Zornik & Yamaguchi, 2012), less is known about the neurochemicals that modulate their activity (e.g., Forlano, Kim, Krzyminska, and Sisneros, 2014; Yu & Yamaguchi, 2010; Zornik, Katzen, Rhodes, and Yamaguchi, 2010). The well‐established neuroanatomical and neurophysiological characterization of the vocal CPG in species such as the Gulf toadfish, *Opsanus beta*, together with the ability to unambiguously identify each vocal CPG nucleus via transneuronal neurobiotin labeling, present a distinct opportunity to investigate the neurochemical profile of each component of an evolutionarily conserved vertebrate vocal CPG (Bass et al., 2008; Chagnaud & Bass, 2014). Here, we report the robust distribution of inhibitory neurotransmitters and neuromodulators within the Gulf toadfish vocal CPG that likely contribute to the ability of these and other species of toadfish to produce social context‐dependent vocal behaviors with divergent temporal and spectral properties (Rice & Bass, 2009).

## 2 MATERIALS AND METHODS

### 2.1 Animals

Seventeen Gulf toadfish, *Opsanus beta*, (12 males, four females, sex not reported for one fish; 3.5–18.7 cm in standard length; median = 10.5 cm; interquartile range = 10.0) were obtained from a commercial source (Gulf Specimen, Panacea, FL) and housed in aquaria at 22°C in an environmental control room on a 12:12 dark:light cycle. All surgical methods and collection of tissues were approved by the Cornell University Institutional Animal Care and Use Committee.

### 2.2 Vocal CPG labeling

Neurobiotin crystals (Vector Laboratories, Burlingame, CA) were applied to the cut end of one vocal nerve at the level of the swim bladder. A detailed description of the methods can be found in Bass et al. (1994). After a survival time of 1.5–7 days following neurobiotin application, fish were deeply anaesthetized by immersion in aquarium water with benzocaine (0.025%; Sigma Aldrich, St. Louis, MO) and then transcardially perfused with 4% paraformaldehyde (PFA) or 3.75% PFA and 0.25% glutaraldehyde in 0.1 M phosphate buffer (PB; all: Sigma Aldrich, St. Louis, MO). Brains were post‐fixed in the same solution for 1 hr at 4°C and stored in 0.1 M PB at 4°C.

### 2.3 Immunohistochemistry (IHC)

One day prior to sectioning, fixed brains were cryoprotected in 30% sucrose in 0.1 M PB (Carl Roth GmbH + Co. KG, Karlsruhe, Germany) at 4°C overnight. Brains were sectioned in the transverse plane with a cryostat at 25 µm (Leica microsystems, Wetzlar, Germany) and directly mounted onto microscope slides (Superfrost Ultra Plus Adhesion Slides; Thermo Fisher Scientific Inc., Braunschweig, Germany). Slides were subsequently left at room temperature for 1 hr to allow the sections to dry and then either processed for IHC immediately or stored at −80°C. Each brain was sectioned into four complete series, each of which was stained with a single antibody.

For IHC, the slides were immersed in 0.1 M PB‐saline (PBS) for 30 min for rehydration. Glutaraldehyde fixed brains were additionally washed in 0.001% sodium borohydride (Sigma Aldrich Chemie GmbH, Munich, Germany) in 1 ml 0.1 M PBS for 5 min to reduce glutaraldehyde background. A washing series (four times, 5 min each) in 0.5% Triton 100 (Sigma Aldrich Chemie GmbH, Munich, Germany) in 0.1 M PBS (PBS‐T) followed. Subsequently, each slide was washed in 10% normal donkey serum (Jackson Immunoresearch Europe Ltd., Suffolk, United Kingdom) in PBS‐T for 1 hr, before incubation with primary antibody (Table 1) overnight. Slides were then washed four times in PBS‐T before incubation with secondary antibody and anti‐biotin antibody (Table 1) for 4 hr. Slides were again washed four times with PBS‐T, dried and then coverslipped using a fluorescent mounting medium (Vectashield, Vector Labs Inc., Peterborough, United Kingdom) containing 4′,6‐diamidino‐2‐phenyindole (DAPI). All incubation and washing steps were performed at room temperature.

**Table 1:** List of primary and secondary antibodies including their name, immunogen, manufacturer, catalogue number, RRID, host, antibody type, the dilution used in this study and fixation type

Antibody name	Immunogen	Manufacturer, Catalogue number, RRID, Host, Antibody type	Dilution	Fixation type
*Primary antibodies*				
ChAT	Human placental enzyme	Millipore, Ab144P, RRID:AB_2079751, goat, polyclonal	1:500	PFA
Connexin 35/36	Recombinant Perch Connexin 35	Millipore, MAB3045, RRID:AB_94632, mouse, monoclonal	1:200	PFA or PFA/GLUT
GABA	GABA coupled to bovineserum albumin	Swant, mAB 3D5, RRID:AB_10013381, mouse, monoclonal	1:200	PFA/GLUT
Glycine	Glycine‐glutaraldehyd‐carriers	MoBiTec, 1015GE, RRID:AB_2560949, rabbit, polyclonal	1:200	PFA/GLUT
Serotonin	Serotonin coupled to bovine serum albumin with paraformaldehyde	Immunostar, 20080, RRID:AB_10718516, rabbit, unknown	1:500	PFA or PFA/GLUT
Tyrosine hydroxylase	TH purified from rat PC12 cells	Immunostar, 22941, RRID:AB_572268, mouse, monoclonal	1:500	PFA or PFA/GLUT
*Secondary antibodies*				
Alexa Fluor antibody	Mouse gamma Immunoglobins Heavy and Light chains	Jackson ImmunoResearch Labs, 715‐545‐150, RRID:AB_2340846, donkey, polyclonal	1:200	PFA or PFA/GLUT
Donkey Anti‐goat IgG (H+L)	Goat IgG (H+L)	Molecular Probes, A11055, RRID:AB_142672, donkey, polyclonal	1:200	PFA or PFA/GLUT
Donkey Anti‐Rabbit IgG (H+L) Antibody, Alexa Fluor 488 Conjugated	Rabbit gamma Immunoglobins Heavy and Light chains	Molecular Probes, A‐21206, RRID:AB_141708, donkey, unknown	1:200	PFA or PFA/GLUT
*Anti‐biotin antibody*				
Cy3‐Streptavidin antibody	Streptavidin	Jackson ImmunoResearch Labs, 016–160‐084, RRID:AB_2337244, donkey, unkown	1:500	PFA or PFA/GLUT

Abbreviations: PFA, 4% paraformaldehyde; PFA/GLUT, 3.75% paraformaldehyde + 0.25% glutaraldehyde.

Images of brain sections were taken on a confocal laser microscope (Leica microsystems, Wetzlar, Germany) and at an epifluorescence microscope (ECLIPSE Ni, Nikon GmbH, Düsseldorf, Germany). Acquired confocal images were stacked and converted to maximum z‐projections using the free software Fiji (Schindelin, Arganda‐Carreras, & Frise, 2012). Maximum z‐projections were cropped, resized and contrast and brightness were optimized for the entire image using Adobe Photoshop CS6 (Adobe Systems Software Ireland Limited, Dublin, Ireland).

### 2.4 Data analysis

For three similar‐sized fish (OB‐15‐05 (ID code): 5.3 cm (standard length); OB‐15‐06: 5.1 cm; OB‐16‐10: 6.1 cm), we evaluated several dimensions of the neurobiotin‐labeled vocal CPG nuclei (VMN, VPN, VPP): rostral‐caudal extent of each nucleus, neuron number for each nucleus, individual neuron size reported as diameter, and individual neuron shape evaluated by the shape factor. Rostral‐caudal extent was determined by counting all sections of each brain where neurons of the respective nuclei were labeled and then multiplied by 25 µm, that is, the section thickness. Neuron number was estimated using the following criteria: First, in one series (out of four, see above) of each brain all neurobiotin labeled neurons were counted in which the nucleus of a given neuron could be identified with DAPI. This value was multiplied by four to account for neurons in the four brain series (see section 2.3), and then corrected using the Abercrombie correction factor (Abercrombie, 1946). For each fish the diameter of the nucleus was measured in 10 neurons of each cell population (VMN, VPN, VPP), and the average for each population was used for the Abercrombie correction for each respective population. Neuronal soma diameter was calculated as the average of minor and major axis measurements in each fish as has been done previously in *O. beta* (Chagnaud & Bass, 2014). Shape factor was calculated as the aspect ratio of a neuron's smallest dimension in a single plane (minor axis) divided by the neuron's largest dimension (major axis). A shape factor of one represents a perfect circle with decreasing values indicating more elongated shapes. Minor and major axes were measured with the ruler tool in Adobe Photoshop CS6 software.

As we report, four groups of labeled neurons were recognized in the premotor VPN region: neurobiotin‐only, GABA‐only, glycine‐only, and neurobiotin‐glycine co‐labeled. A non‐neurobiotin labeled neuron (i.e., glycinergic or GABAergic) was attributed to a vocal CPG nucleus if it was surrounded by neurobiotin‐labeled neurons of that nucleus or if it was located adjacent to such neurons. Due to the heterogeneity of VPN neurons, neuron number and diameter were assessed separately for those groups along with the neuron shape factor. In order to avoid size effect between fishes, neuron diameter and shape factor were compared between the VPN groups in three fish (OB‐15‐04: 10.5 cm; OB‐16‐09: 8.5 cm; OB‐16‐10: 6.1 cm) that exhibited neurobiotin, GABA and glycine label. For each fish, we separately tested if neuron diameter and shape factor were significantly different between the four neuron groups. We visually assessed a normal distribution for each neuron group within each fish using normal quantile–quantile plots (Supporting Information Figure S1). We used a Kruskal‐Wallis test with a Wilcoxon signed rank test as a post‐hoc test only if the Kruskal‐Wallis test gave a significant result. The *p*‐value was adjusted according to a conservative Bonferroni correction which gave 0.008 as new level of significance for posthoc test results. All values are given as median with the interquartile range as a measure of variability. All calculations and tests were performed in R (R Core Team, 2014, version 3.14).

To identify a potential topographic organization of the different neuron types in VPN, we investigated their respective distribution by overlaying all sections of a single, ipsilateral population of VPN neurons from one fish (OB‐15‐04). For each VPN containing hindbrain section, we marked each neuron type with an asterisk or star (see Figure 5a, b). Next, we formed a polygon by connecting neurobiotin‐positive neurons with lines that enclose all other neurobiotin‐positive neurons in one section. We restricted this step to neurobiotin‐positive neurons because they form the principal neurons of VPN. For the resulting polygon, we calculated the centroid using the python script “Centroid.py” provided by Robert Fotino (https://gist.github.com/rfotino/a0fa1ef2882484e2da89#file-centroid-py). With the calculated centroids for each section, we aligned all VPN sections to construct a representation of the neurons within the VPN region by marking each neuron individually with a color coded cross and by displaying the total area covered by each neuron group (Adobe Photoshop).

### 2.5 DAB staining

Two reference brains from animals with transneuronal, neurobiotin‐labeling of the vocal CPG were visualized with the VECTASTAIN ABC HRP Kit followed by the 3,3′‐diaminobenzidine (DAB) substrate kit (both: Vector Labs Inc., Peterborough, United Kingdom). Briefly, sections were washed in distilled water for 5 min followed by incubation in 0.3% hydrogen peroxidase in 70% methanol to remove endogenous peroxidase from the tissue. Sections were then incubated in VECTASTAIN ABC reagent for 2 hr. Afterwards, the sections were washed with 0.1 M PB for 5 min and incubated with DAB substrate kit working solution for 2–10 min followed by three 5 min washing steps with 0.1M PB. Slides were counterstained with cresyl violet and washed in 70% and 96% (2×) ethanol followed by 100% iso‐propanol (2×) and xylol (3×). Each washing step lasted 3 min. Lastly, slides were coverslipped with Roti‐Histokit II (Carl Roth GmbH + Co. KG, Karlsruhe, Germany) and images taken in the brightfield channel of an epifluorescence microscope (ECLIPSE Ni, Nikon GmbH, Düsseldorf, Germany).

### 2.6 Antibody characterization

#### 2.6.1 GABA

We used a monoclonal antibody (mAB 3D5; Swant, Marly, Switzerland; RRID:AB_10013381) raised in mouse to detect gamma‐aminobutyric acid (GABA). Antibody specificity was determined by the manufacturer in an enzyme linked immunosorbent assay (ELISA) by testing cross‐reactivity with β‐alanine, aspartate, glutamate, glycine, and taurine. This revealed low cross‐reactivity. This antibody was previously used to identify the presence of GABA in sea lamprey, *Petromyzon marinus* (Villar‐Cerviño et al., 2006).

#### 2.6.2 Glycine

A polyclonal antibody (1015GE; MoBiTec, Göttingen, Germany; RRID:AB_2560949) raised in rabbit was used to identify the amino acid glycine. Antibody specificity was tested by the manufacturer with a glycine‐glutaraldehyde‐protein in an ELISA test by cross‐reactivity experiments with β‐alanine, aspartate, GABA, glutamate, and taurine. This showed low cross‐reactivity. The antibody used here was previously used to identify glycinergic neurons in the auditory and vestibular system in guinea pig, *Cavia porcellus* (Peyret, Campistron, Geffard, & Aran, 1987) and in the brain of rats, *Rattus rattus* (Campistron, Buijs, & Geffard, 1986) with no trace of glial labeling. Other studies have used antibodies against glycine to identify glycinergic neurons without any sign of glial labeling in mouse (Restrepo et al., 2009), rat (Downie et al., 2010), sea lamprey (Villar‐Cerviño, Barreiro‐Iglesias, Anadón, & Rodicio, 2008), Siberian sturgeon, *Acipenser baeri* (except for coronet cells) (Adrio, Rodríguez‐Moldes, & Anadón, 2011) and salamander, *Ambystoma tigrinum* (Cimini, Strang, Wotring, Keyser, & Eldred, 2008).

#### 2.6.3 Connexin

**Figure 2:**
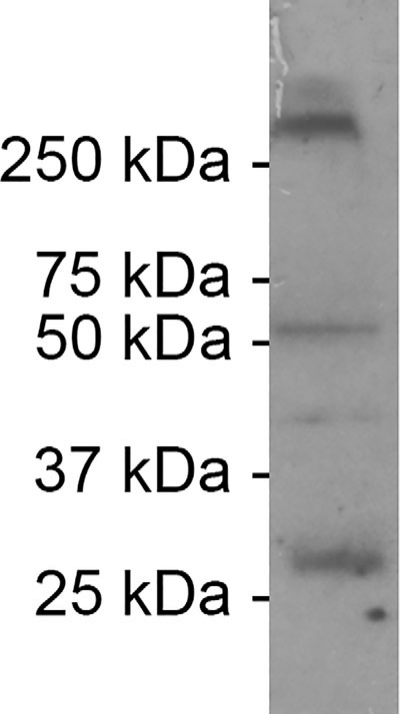
Connexin 35/36 antibody labeling in western blot of whole brain homogenate of Gulf toadfish. Bands are between 25 and 37 kilo Dalton (kDa) ladder marks, as well as between the 50 and 75 kDa ladder marks. Results are identical to the antibody manufacturer's results in hybrid bass (exact species not provided on Millipore website for antibody MAB3045), a different fish species than the one toward which the antibody was raised. The heavy band above the 250 kDa ladder mark likely represents the six connexin protein subunits binding together to form a connexin hemichannel

A monoclonal antibody (MAB3045; Millipore, Bedford, MA; RRID:AB_94632) raised in mouse was used to detect connexin 35/36 (Cx 35/36), a pore protein in gap junctions. The specificity assessment of the manufacturer states that this antibody reacts with fish Cx 35/36. The antibody has been used to stain Cx 35/36 in zebrafish, *Danio rerio* (Song, Ampatzis, Björnfors, & El Manira, 2016) where it was shown to be present in electrical synapses between motor and premotor neurons, similar to the VMN‐VPN‐VPP coupling in this study. We tested the antibody's specificity using a western blot of Gulf toadfish whole brain homogenates. 12% resolving, 5% stacking SDS‐polyacrylamide gel electrophoresis was performed on whole brain homogenates. Gels were transferred to polyvinylidene difluoride membrane (Bio‐Rad Laboratories Inc., Hercules, CA), blocked overnight at 4°C in PBS with 10% powdered milk, and then overnight at 4°C in PBS with 10% powdered milk with 1:1,000 primary antibody against Cx 35/36. Subsequently, gels were washed with PBS and incubated in secondary antibody for 2 hr at room temperature. After washing in PBS, gels were treated with a chemiluminescence kit (SuperSignal West Pico; Thermo Scientific Waltham, MA), and exposed to film (Carestream Kodak BioMax MS; Sigma Aldrich, St. Louis, MO). The film was developed and scanned as 600 dpi grayscale tiff files (Epson Perfection 4990 Photo scanner; Epson, Long Beach, CA). The antibody manufacturer reports two bands on western blots of hybrid bass whole brain (exact species not provided on Millipore website): one between 25 and 37 kDalton (kDa) and one between 50 and 75 kDa. We found similar results in Gulf toadfish whole brain homogenates on western blots (Figure 2). An additional band >250 kDa is likely the result of six connexin protein subunits coming together to form a single connexin hemi‐channel.

#### 2.6.4 Serotonin

A polyclonal antibody against serotonin (20080; Immunostar, Hudson, WI; RRID:AB_10718516) raised in rabbit was used to detect serotonin. According to the manufacturer, this antibody is specific toward its target in a variety of vertebrate species and has been used in studies of the Atlantic salmon, *Salmon salar*, (Sandbakken, Ebbesson, Stefansson, & Helvik, 2012), and sea lamprey (Villar‐Cerviño et al., 2006). Additionally, the manufacturer states that the antibody serum does not react with 5‐hydroxytryptophan, 5‐hydroxyinodole‐3‐acetic acid and dopamine in cross‐reactivity experiments.

#### 2.6.5 Tyrosine hydroxylase

We used a monoclonal antibody that was raised in mouse against tyrosine hydroxylase (TH; 22941; Immunostar, Hudson, WI; RRID:AB_572268), the rate‐limiting enzyme in catecholamine synthesis, to detect catecholaminergic input to the vocal CPG. This antibody detects the catalytic core of TH and therefore reacts with TH in a wide range of species including amphibians (Joven, Morona, González, & Moreno, 2013) and zebrafish (manufacturer's statement). The antibody does not react with phenylalanine hydroxylase or tryptophan hydroxylase (manufacturer's statement). Western blot analysis of brain extract of midshipman, a close relative of Gulf toadfish, showed a band at 59–63 kDa as expected for TH according to the manufacturer (Goebrecht, Kowtoniuk, Kelly, & Kittelberger, 2014).

#### 2.6.6 Choline acetyltransferase

A polyclonal antibody raised in goat against choline acetyltransferase (ChAT, AB144P; Millipore, Bedford, MA; RRID:AB_2079751), the rate limiting enzyme in the synthesis of acetylcholine (ACh), was used to assess the presence of ACh. This antibody has been tested in a wide range of species including zebrafish (Müller, Vernier, & Wullimann, 2004), goldfish, *Carassius auratus* (Giraldez‐Perez, Gaytan, & Pasaro, 2013), bichir, *Polypterus senegalus* (Lopez, Perlado, Morona, Northcutt, & Gonzalez, 2013) and weakly electric mormyrid fish, *Gnathonemus petersii* (Pusch, Wagner, von der Emde, & Engelmann, 2013). In agreement with the manufacturer's western blot analysis in mouse brain lysate, the antibody was shown to specifically recognize bands between 68 and 72 kDa, for example, in zebrafish (Coppola, D'autréaux, Nomaksteinsky, & Burnet, 2012), the lesser spotted dogfish, *Scyliorhinus canicula* (Anadón et al., 2000), and bichir (Lopez et al., 2013), corresponding to the size of ChAT protein in zebrafish (70 kDa; Volkmann, Chen, Harris, Wullimann, and Koster, 2010).

For all primary and secondary antibodies, we tested for nonspecific binding of the antibody to the tissue by carrying out the staining protocol as above but omitting the secondary or primary antibody, respectively. All tests revealed no staining.

## 3 RESULTS

### 3.1 Identification of the vocal CPG nuclei

Confirming the results of a prior study of Gulf toadfish (Chagnaud & Bass, 2014), transneuronal neurobiotin transport after neurobiotin application to the VN (Figure 1e) labels the three vocal CPG nuclei—VPP, VPN, and VMN (Figure 1e–h). In contrast, applying crystals of the much larger molecular weight biotinylated dextran (3 kDa compared to 323 Da for neurobiotin) leads only to a unilateral label of the VMN (Chagnaud & Bass, 2014). The paired VMN form a single dense column of motor neurons with the paired VPN extending alongside as ventrolateral columns. VPP appears bilaterally as ventrolateral columns just rostral to VMN and VPN. Based on measurements in three animals, the rostral‐caudal extent, number, diameter, and shape factor of neurobiotin‐labeled neurons in the three CPG nuclei (Table 2) is consistent with values reported in a previous study (Chagnaud & Bass, 2014).

**Table 2:** Rostro‐caudal nucleus extent [µm], neuron number (Abercrombie corrected), neuron diameter [µm] and shape factor of neurobiotin‐labeled neurons evaluated bilaterally in all three nuclei of the vocal CPG. All values are average and standard deviation in parentheses

	VMN	VPN	VPP
Rostro‐caudal extent [µm]	983 (154)	800 (20)	308 (72)
Neuron number	948 (133)	375(52)	224 (15)
Neuron diameter [µm]	17 (2)	10 (1)	10 (1)
Neuron shape factor	0.744 (0.119)	0.805 (0.105)	0.762 (0.116)

Abbreviations: VMN, vocal motor nucleus; VPN, vocal pacemaker nucleus; VPP, vocal pre‐pacemaker nucleus.

### 3.2 GABAergic and glycinergic label in vocal CPG

**Figure 3:**
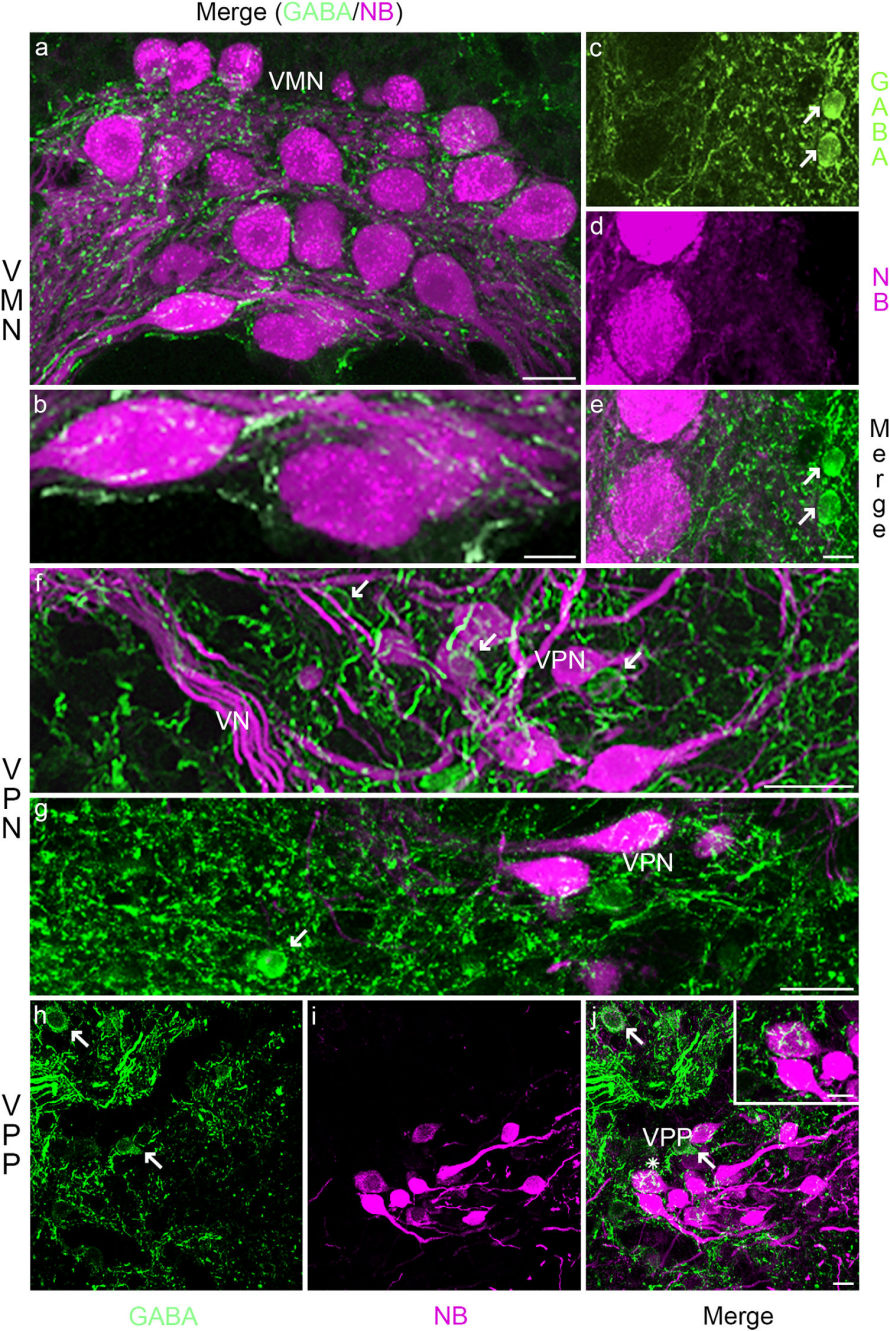
GABA in Gulf toadfish vocal CPG. GABAergic label (green) is present in all of the neurobiotin labeled CPG nuclei (NB; magenta): (a–e) vocal motor nucleus (VMN), (f, g) vocal pacemaker nucleus (VPN), (h–j) vocal pre‐pacemaker nucleus (VPP). GABAergic somata are observed next to the VMN (arrows in c, e); next to and within the VPN (arrows in f, g) and VPP (arrows in h, j). Asterisk in (j) indicates somata in inset. GABAergic neurons are not co‐labeled with neurobiotin (e.g., c–e; f, g; h–j). The scale bar represents 25 µm in (a), 10 µm in (b), 10 µm in (e) for (c–e), 10 µm in (f) and (g), 10 µm in (j) for (h‐j) and 5 µm in inset in (j). Abbreviations: VN, vocal nerve tract

Neurophysiological evidence supports the role of GABA in determining the temporal properties of the vocal CPG output (Chagnaud et al., 2011; Chagnaud et al., 2012). Dense GABAergic label is present at all levels of the vocal CPG (Figure 3). Labeled puncta suggestive of synaptic sites occur on neurobiotin‐labeled somata or within the adjacent neuropil of all three vocal nuclei (Figure 3a, b, e, f, g, j). To identify the putative origin of these inputs, we looked for somata that were located immediately adjacent or close to neurobiotin‐labeled neurons. Small GABAergic somata, round in shape, are lateral to the VMN (arrows, Figure 3c, e); dorsal, ventral and lateral to VPN (e.g., arrow indicates ventrally located GABAergic neurons, Figure 3g), as well as within VPN (arrows, Figure 3f); and within and around VPP (arrows, Figure 3h, j).

**Figure 4:**
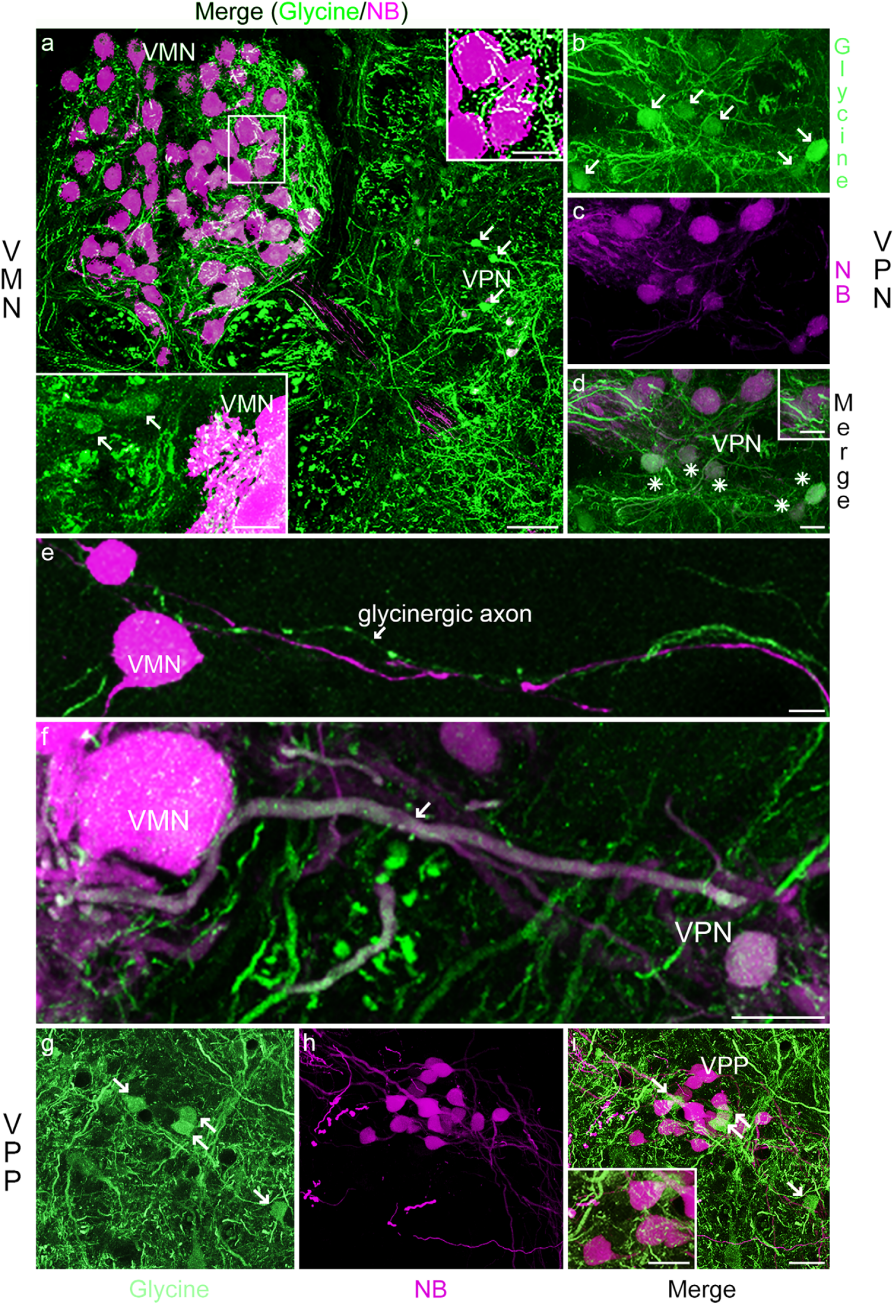
Glycine in Gulf toadfish vocal CPG. Glycinergic label (green) lies within all of the neurobiotin‐labeled CPG nuclei (NB; magenta): vocal motor (VMN, a), vocal pacemaker (VPN; b–d), vocal pre‐pacemaker (VPP; g,i). Glycinergic somata are observed next to VMN (arrows, lower inset in a), and within VPN (arrows in a, b) and VPP (arrows in g, i). A subset of glycinergic neurons in the VPN are co‐labeled with neurobiotin (asterisks in d). The upper right inset in (a) is higher magnification view of VMN. The inset in (i) highlights glycinergic label in VPP. A glycine‐labeled axon in close proximity to a VMN soma is highlighted in (e). Neurobiotin‐glycine co‐labeled processes (f, arrow) are observed between VPN and VMN. The scale bar represents 50 µm in (a), 10 µm in (d) for (b–d), 20 µm in (e), 10 µm in (f), and 10 µm in (i) for (g–i). The scale bar is 25 µm in the upper right inset in (a), 10 µm in the lower left inset in (a) and 10 µm in insets in (d) and (i)

Similar to GABAergic label, glycinergic label is present in all vocal CPG nuclei (Figure 4), including puncta suggestive of synapses (Figure 4a, d, i and insets). Glycinergic neurons with round‐like somata are adjacent and lateral to VMN (arrows, Figure 4a, lower left inset), and adjacent and within VPN (e.g., arrows, Figure 4a, b) and VPP (arrows, Figure 4g, i). In contrast to GABAergic neurons, a subset of glycinergic neurons are co‐labeled with neurobiotin within VPN (Figure 4b–d, see asterisks in d). Neurobiotin‐glycine, co‐labeled fibers also enter VMN (arrow, Figure 4f). There are no co‐labeled somata or processes in VPP (Figure 4g–i, see arrows in g, i).

### 3.3 Subsets of inhibitory neurons within VPN

VPN has four types of labeled neurons: neurobiotin‐only, GABA‐only, glycine‐only, and neurobiotin‐glycine co‐labeled. To assess if these neurons form distinct subpopulations, we compared the proportion of neurons, and the neuron diameter and shape factor in each of the four VPN groups in three fish (see section 2.4). In these fish, neurobiotin, glycine and GABA are co‐stained. To avoid size effects due to different body sizes, statistical tests were performed only within individual fish. The results are summarized in Table 3. Table 3a presents a summary of the results for neuron diameter and shape in each fish; Table 3b,c present the statistical results for these measures for each fish in which significant differences were observed. The majority of neurons are only labeled with either neurobiotin or glycine. Fewer neurons are labeled with only GABA or with both neurobiotin and glycine.

**Table 3:** a) Median and interquartile range (in parentheses) of neuron dimensions in subgroups of the vocal pacemaker nucleus (VPN) in three fish (animal ID code and standard length indicated) assessed as diameter [µm] and shape factor. Results of Kruskal‐Wallis test and Wilcoxon signed rank test for differences in neuron diameter (b) and shape factor (c) between neuron groups in VPN

a) Neuron dimension and shape factor					
		NB‐only	Glycine‐only	NB‐Glycine	GABA‐only
**Toadfish 1**	diameter [µm]	13 (4)	10 (3)	11 (3)	8 (2)
OB‐15‐04 (10.5 cm)	Shape factor	0.704(0.216)	0.709 (0.229)	0.690 (0.177)	0.792 (0.173)
**Toadfish 2**	diameter [µm]	12 (3)	11 (2)	12 (2)	8 (2)
OB‐16‐09 (8.5 cm)	Shape factor	0.769 (0.183)	0.770 (0.134)	0.799 (0.2)	0.780 (0.232)
**Toadfish 3**	diameter [µm]	11 (3)	10 (2)	9 (3)	8 (2)
OB‐16‐10 (6.1cm)	Shape factor	0.789 (0.131)	0.803 (0.157)	0.752 (0.139)	0.824 (0.232)

b) Neuron diameter			
Kruskal‐Wallis test	Chi^2^	Degrees of freedom	*p*‐value
Toadfish 1 (OB‐15‐04)	94.883	3	**<.0001**
Toadfish 2 (OB‐16‐09)	65.576	3	**<.0001**
Toadfish 3 (OB‐16‐10)	33.811	3	**<.0001**

Wilcoxon signed rank test							
		Gly‐only		NB‐Gly		GABA‐only	
		*W*	*p*	*W*	*p*	*W*	*p*
**Toadfish 1**	NB‐only	3567	**<.0001**	3254	**.0003**	3240.5	**<.0001**
(OB‐15‐04; 10.5cm)	Gly‐only			1731.5	.341	1905	**<.0001**
	NB‐Gly					1999.5	**<.0001**
**Toadfish 2**	NB‐only	905	**.0010**	239	.0187	956	**<.0001**
(OB‐16‐09; 8.5cm)	Gly‐only			191	.1281	1109	**<.0001**
	NB‐Gly					300	**<.0001**
**Toadfish 3**	NB‐only	3480.5	**.0023**	1216	**.0023**	1185	**<.0001**
(OB‐16‐10; 6.1cm)	Gly‐only			274	.1204	507	**<.0001**
	NB‐Gly					118	.1828

c) Shape factor			
Kruskal‐Wallis test	Chi^2^	Degrees of freedom	*p*‐value
Toadfish 1 (OB‐15‐04)	12.403	3	**.0061**
Toadfish 2 (OB‐16‐09)	1.8371	3	.6069
Toadfish 3 (OB‐16‐10)	0.4232	3	.9354

Wilcoxon signed rank test							
		Gly‐only		NB‐Gly		GABA‐only	
		*W*	*p*	*W*	*p*	*W*	*p*
**Toadfish 1**	NB‐only	2367	.5695	2484	.7922	1101	**.0013**
(OB‐15‐04, 10.5cm)	Gly‐only			1438	.4528	648	**.0011**
	NB‐Gly					799	.0199

*Note*. Highly significant results are marked in bold.

Abbreviations: NB, neurobiotin; Gly glycine

The neurobiotin‐only labeled neurons are, in general, significantly larger in diameter than glycine‐only labeled and neurobiotin‐glycine co‐labeled neurons (*p* < .02 in all fish; Table 3b). Glycine‐only and neurobiotin‐glycine labeled neurons have a similar diameter. GABA‐labeled neurons are significantly smaller in diameter than neurobiotin‐only, neurobiotin‐glycine co‐labeled, and glycine‐only labeled neurons (Table 3b; *p* < .0001 for all comparisons), except for Toadfish 3 that shows no significant difference in diameter between GABA‐only labeled and neurobiotin‐glycine co‐labeled neurons (Table 3b). The shape factor of GABA‐only neurons is significantly different from neurobiotin‐only and glycine‐only neurons, but not from neurobiotin‐glycine co‐labeled neurons in only the largest of the three toadfish examined (Table 3c). While this might suggest an effect of fish size on neuronal dimensions, a much larger sample size would be needed to assess this possibility.

**Figure 5:**
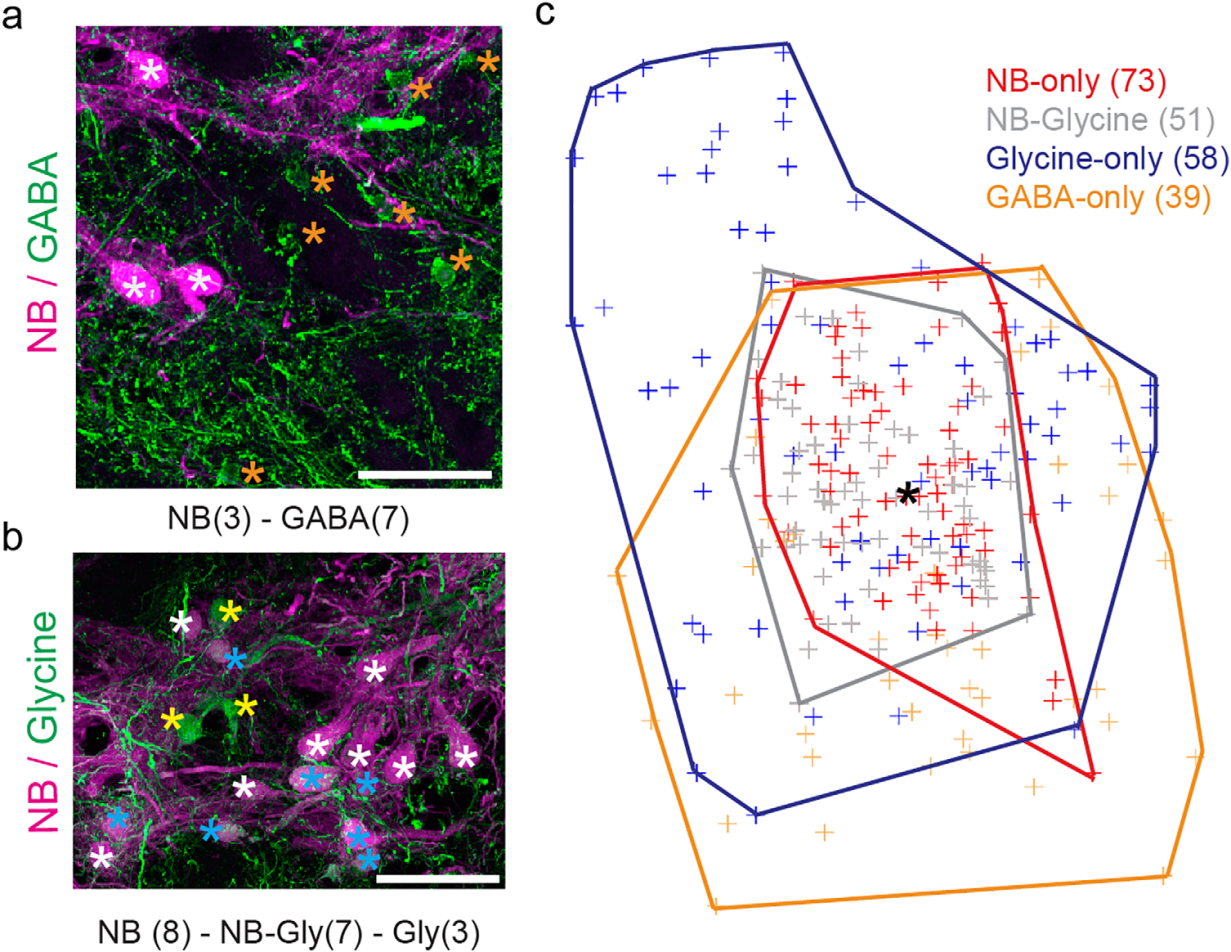
Distribution of GABAergic and glycinergic neurons in vocal pacemaker nucleus (VPN) of Gulf toadfish. Representative photomicrographs (a, b) of a single, ipsilateral VPN population in which the position (asterisks) of neurobiotin (NB; white asterisks in (a) and (b)), GABA (orange asterisks in (a)), glycine‐only (yellow asterisks in (b)) and neurobiotin‐glycine co‐labeled (light blue stars in (b)) neurons are indicated. Sample size is given in parentheses for each group. Color‐coded representations of the distribution of the four neuron groups in the VPN region is shown in (c). Each neuron's location is represented by a color coded cross and the outline of the population by a color‐matched polygon. Sample size is given in parentheses for each group. Black asterisk marks the center of VPN

To determine whether the four VPN neuron groups display a topographic organization, we assessed their distributions throughout the rostral‐caudal extent of VPN in one fish (Figure 5a, b). Neurobiotin‐glycine co‐labeled neurons strongly overlap the distribution of neurobiotin‐only labeled neurons (Figure 5c). The GABA‐only and glycine‐only labeled neurons are predominantly located along and outside the main perimeter of neurobiotin labeled VPN neurons (Figure 5c). Thus, while a spatial separation exists between VPN neurons labeled with or without neurobiotin (neurobiotin‐glycine and neurobiotin‐only versus GABA‐only and glycine‐only, respectively), a neuron's location is not predictive of its labeling pattern.

### 3.4 Gap junction label in vocal CPG

**Figure 6:**
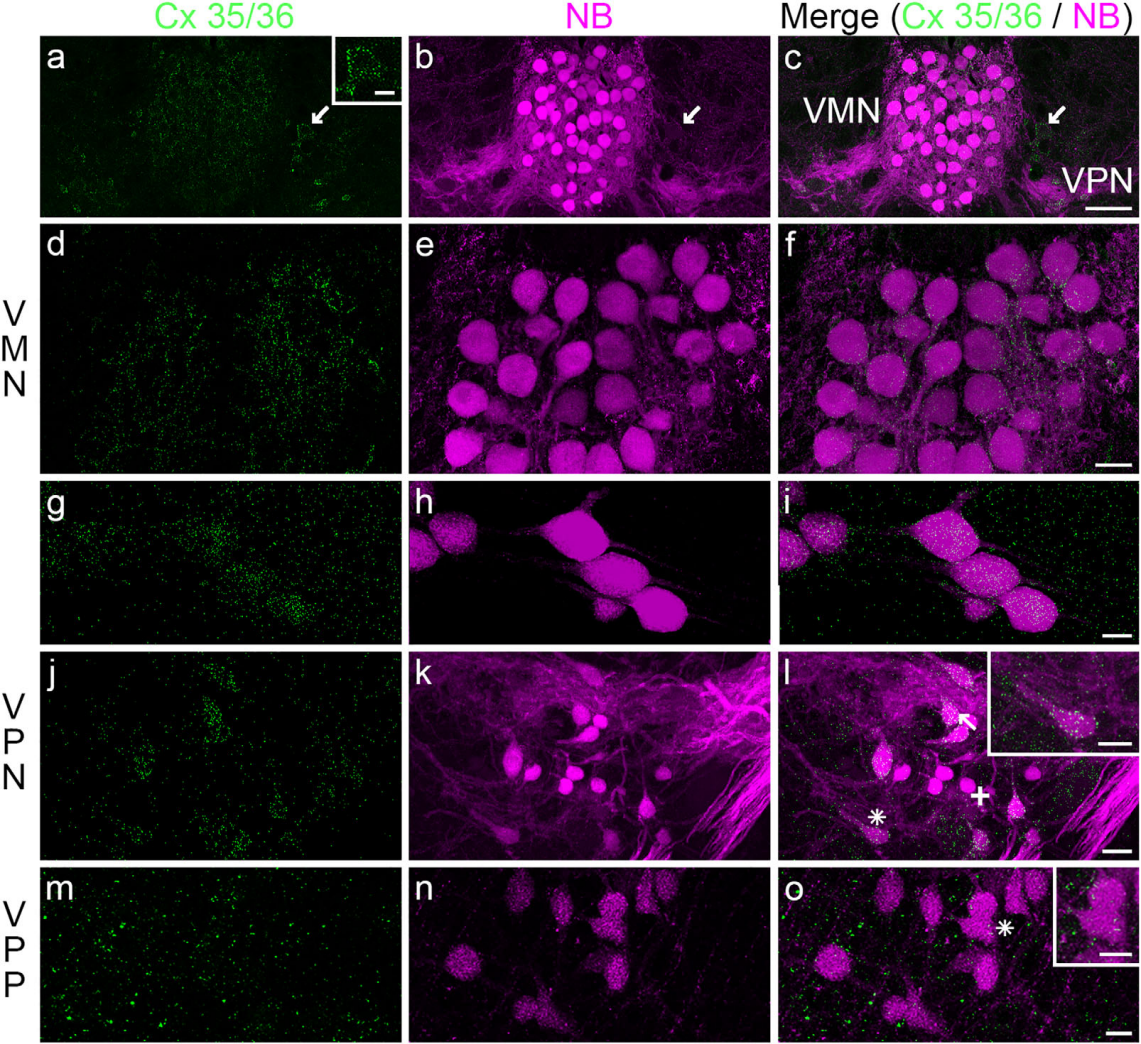
Connexin 35/36 distribution in Gulf toadfish vocal CPG. Connexin 35/36 (Cx 35/36; green) as a marker of gap junctions is present in all CPG nuclei, labeled with neurobiotin (NB; magenta). Cx 35/36 label is prominent on all somata of the vocal motor nucleus (VMN; a–i). Punctate‐like Cx 35/36 label also outlined the perimeter of cells adjacent to VMN that are not co‐labeled with neurobiotin, but similar in shape to VMN neurons (arrow in c). Cx 35/36 label is also prominent on somata of the vocal pacemaker nucleus (VPN; j–l, arrow and cross in l indicate examples of labeled and unlabeled somata, respectively). Asterisk in (l) indicates neuron in inset. Cx 35/36 is also present in the vocal pre‐pacemaker (VPP, m–o and inset in o). Asterisk in (o) indicates neurons in inset. Scale bar represents 100 µm in (c) for (a–c), 25 µm in (f) for (d–f), 10 µm in (i) for (g–i), 10 µm in (l) for (j–l), 10 µm in (o) for (m–o), 20 µm for the inset in (a) and 10 µm for the insets in (l) and (o)

Anatomical and neurophysiological evidence has led to the proposal that the robust transneuronal transport of neurobiotin throughout the vocal CPG depends, in whole or in part, on electrotonic coupling within the VPP–VPN–VMN network (Bass & Marchaterre, 1989; Bass et al., 1994; Chagnaud et al., 2011; Chagnaud et al., 2012). We investigated the distribution of gap junction proteins throughout the vocal CPG using an antibody against Cx 35/36. All neurobiotin‐labeled VMN (Figure 6a–i) and many VPN (Figure 6j–l) somata appear co‐labeled with Cx 35/36. Label in VMN is mainly along the perimeter of somata and processes (Figure 6a–f), except at its rostral end where it is dense over somata (Figure 6g–i). Label in VPN (Figure 6j–l) is also dense over somata (arrow, Figure 6l), though extensive label is also present in regions surrounding these somata (Figure 6l). VPP exhibits diffuse Cx 35/36 label (Figure 6m–o) mainly adjacent to neurobiotin‐labeled somata (Figure 6o). As in VMN (see above), label in VPN (e.g., inset, Figure 6l) and VPP (e.g., inset Figure 6o) has a punctate‐like appearance. A few somata immediately adjacent to VMN that are not labeled with neurobiotin also have a ring of punctate, Cx 35/36‐like immunoreactivity along the outer margin (e.g., arrow in Figure 6a–c).

### 3.5 Serotonergic label in vocal CPG

**Figure 7:**
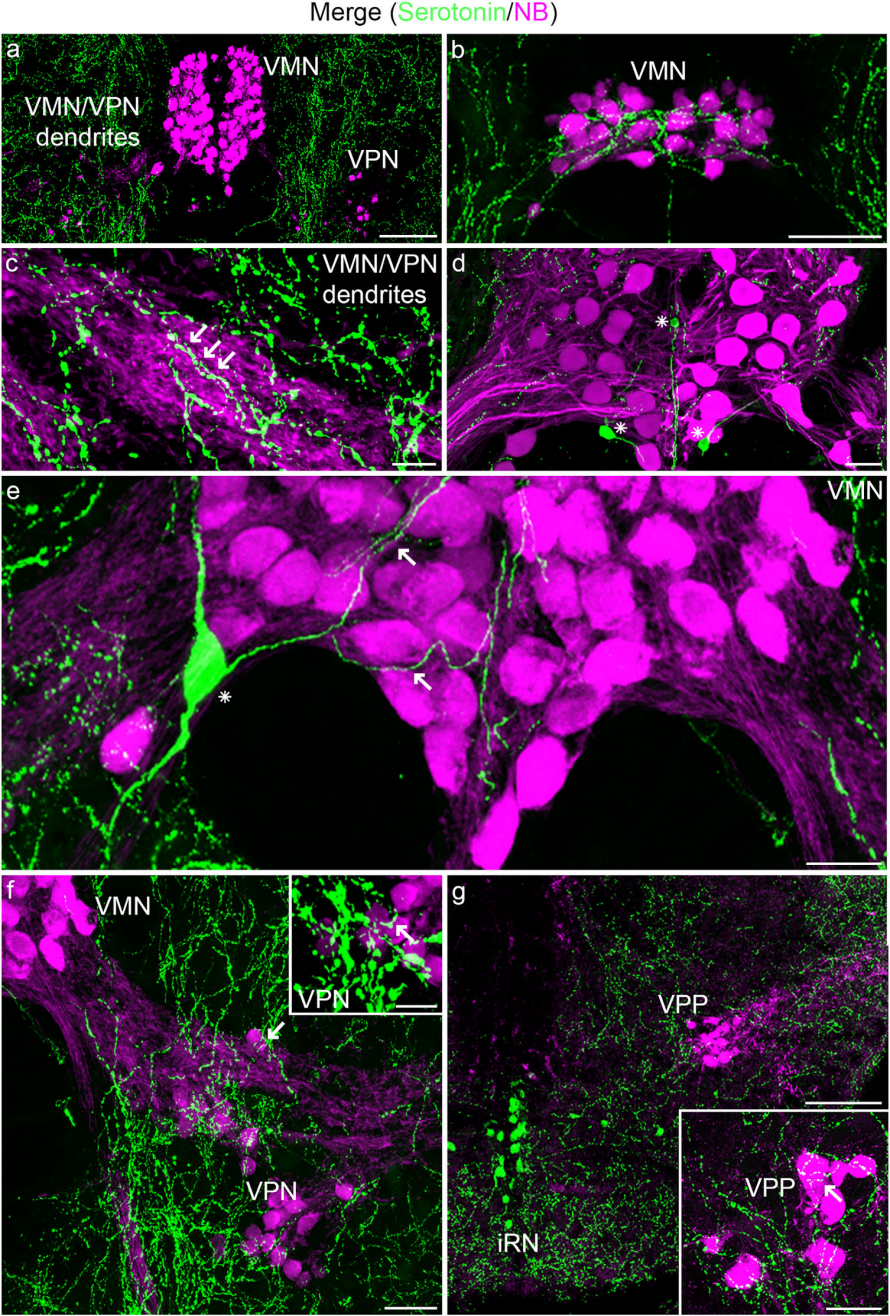
Serotonin in Gulf toadfish vocal CPG. All CPG nuclei, labeled with neurobiotin (NB; magenta), exhibit serotonergic label (green). Weak label is found within most of the VMN (a), except for the rostral pole where it is prominent (b). Serotonergic label is abundant lateral to the VMN, that is, amongst VMN dendrites (arrows in c). Serotonergic somata are within and adjacent to VMN (asterisks in d and e). Serotonergic label in vocal pacemaker (VPN) and pre‐pacemaker (VPP) nuclei is within the region where somata cluster (e.g., arrows in f and insets in f–g). Serotonergic cells are also within the inferior raphe nucleus (iRN) that had a rostral‐caudal extent spanning all levels of the vocal CPG (e.g., at the level of VPP in g). Scale bar represents 100 µm in (a), (b), and (g); 5 µm in (c); 20 µm in (d) and (e); 25 µm in (f); 10 µm in the insets in (f) and (g)

Serotonin modulates the activity of a variety of motor systems including those responsible for vocal behavior (e.g., Wood, Lovell, Mello, and Perkel, 2011; Yu & Yamaguchi, 2010). Serotonergic innervation occurs throughout the vocal CPG (Figure 7). Label in the VMN is minimal (Figure 7a), except for the most rostral pole where it is prominent (Figure 7b). Serotonergic label is abundant lateral to the VMN, amongst the VMN and VPN dendrites (e.g., arrow Figure 7c; see Figure 1f for orientation). In contrast to most of the VMN, label is found throughout VPN and VPP; in some cases, the label is directly over somata (insets, Figure 7f, g), although it is most prominent in adjacent regions (Figure 7f, g).

A few serotonergic cells lie within the central region of VMN (Figure 7d) and along its ventral margin where neurites that branch within VMN are especially apparent (Figure 7e). No serotonergic cells are found within VPN or VPP. None of the serotonergic neurons are co‐labeled with neurobiotin, suggesting that they are not coupled via gap junctions within the vocal CPG. The origin of the serotonergic input to VMN arises, at least in part, from axons of serotonergic cells within and along the perimeter of VMN (Figure 7d, e). The origin of the input to VPN and VPP is less obvious though some input to VPN may come from nearby VMN‐associated serotonergic neurons. Other serotonergic neurons lie further ventral to VMN, VPN, and VPP, or ventral to the fourth ventricle at caudal levels of the cerebellum in the location of the caudal component of the inferior raphe nucleus (iRN, Figure 7g).

### 3.6 Catecholamine label in vocal CPG

**Figure 8:**
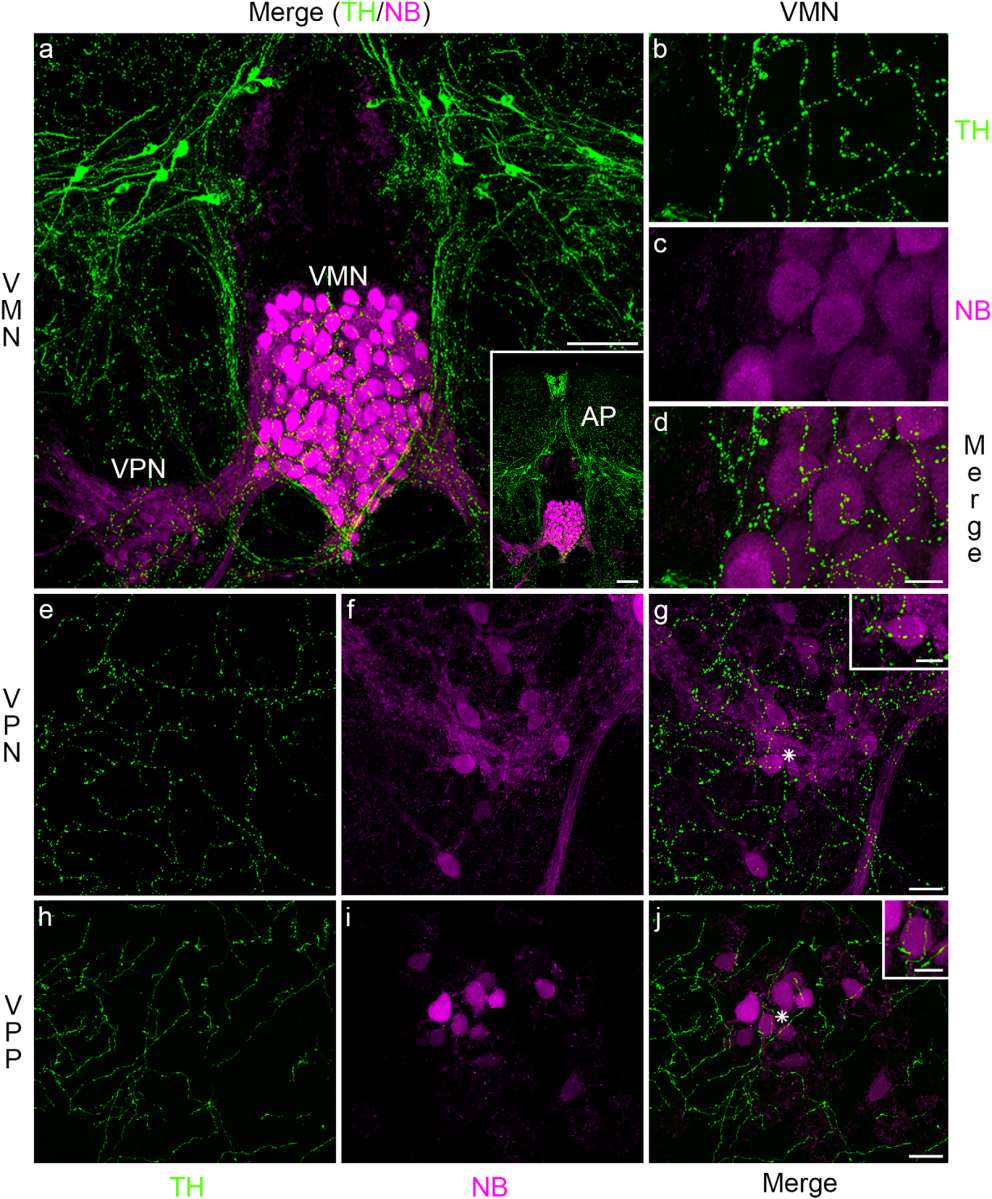
Catecholamines in Gulf toadfish vocal CPG. Dense tyrosine hydroxylase (TH)‐like immunoreactivity indicative of catecholamines (green) occurs within the neurobiotin‐labeled (NB, magenta) vocal motor (VMN, a–d), vocal pacemaker (VPN, e–g) and vocal pre‐pacemaker (VPP, h–j) nuclei. Insets in (g) and (j) highlight catecholaminergic label at sites indicated by asterisks in VPN and VPP, respectively. Catecholaminergic processes likely originate from somata dorsal to the vocal CPG (a) and the area postrema (AP, inset in a). Scale bar represents 100 µm in (a) and its inset, 20 µm in (d) for (b–d), 20 µm in (g) for (e–g), 10 µm in (j) for (h–j), and 5 µm in the insets in (g) and (j)

Given the evidence for a role of catecholamines in vocal mechanisms in several vertebrate taxa (Appeltants, Ball, & Balthazart, 2003; Creighton, Satterfield, & Chu, 2013; Forlano et al., 2014; Goebrecht et al., 2014), we investigated the presence of catecholaminergic input to the vocal CPG using an antibody generated against TH, the rate‐limiting enzyme in the synthesis of dopamine and noradrenaline (Figure 8). All three vocal CPG nuclei exhibit dense TH label (Figure 8a, d, g, j). In contrast to serotonin, catecholaminergic label is abundant both within and lateral to the VMN, VPN, and VPP (Figure 8a–j). Unlike serotonin, there is no apparent rostral‐caudal difference in the intensity of label in VMN. Catecholaminergic neurons are not located within any of the vocal CPG nuclei, although some are dorsolateral to the VMN–VPN circuit (Figure 8a) immediately ventral to the tightly clustered TH cells of the area postrema (Figure 8 inset in a, see also Forlano et al., 2014 for detailed description in midshipman fish). As with serotonin, none of the catecholaminergic neurons are transneuronally labeled with neurobiotin.

### 3.7 Cholinergic label in vocal CPG nuclei

**Figure 9:**
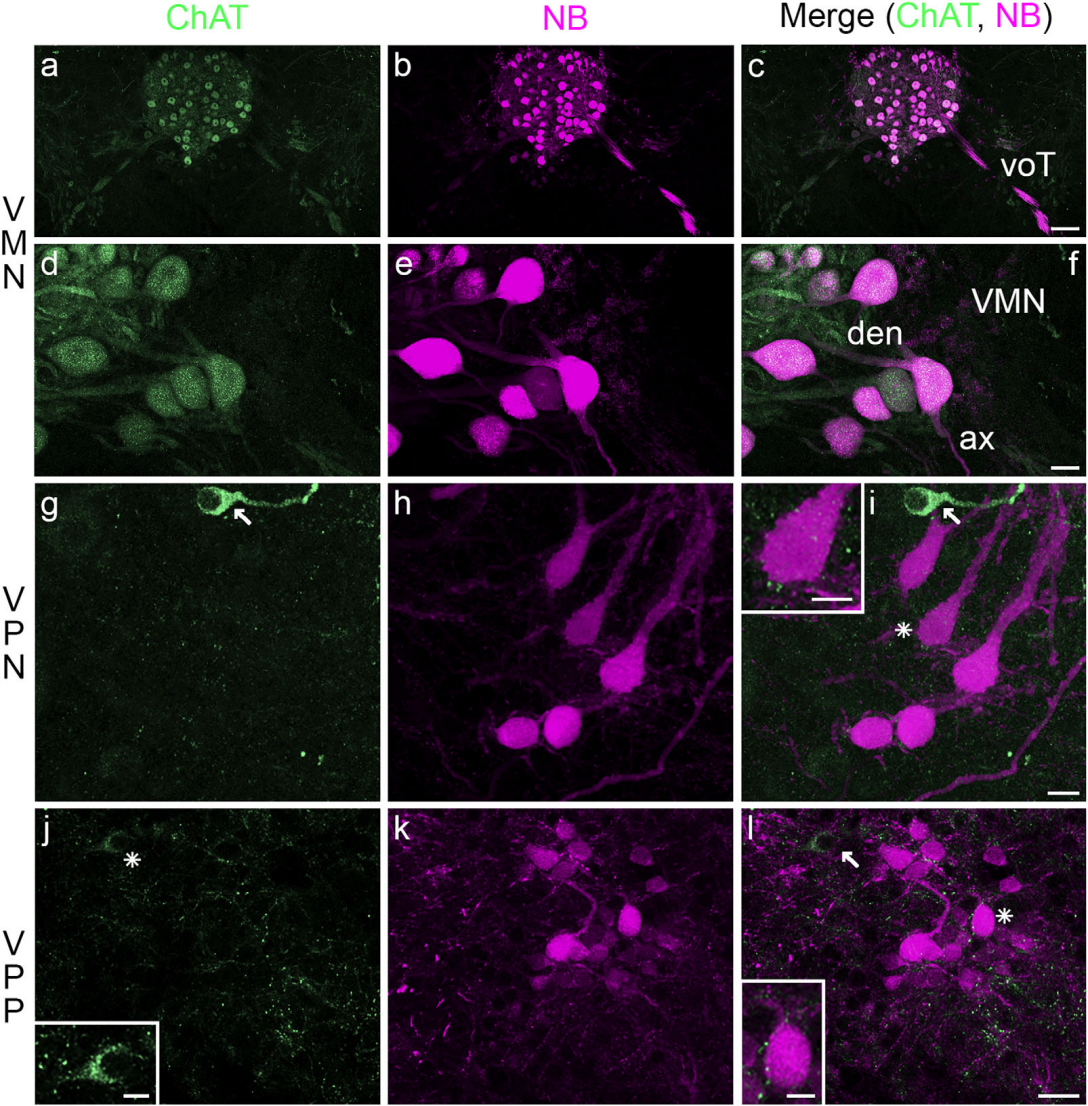
Choline acetyltransferase in Gulf toadfish vocal CPG. Vocal motor nucleus (VMN, a–f) labeled with neurobiotin (NB; magenta) as well as the vocal tract (voT) show choline acetyltransferase (ChAT; green)‐like immunoreactivity (a–f). ChAT label is also present in the vocal pacemaker (VPN, g–i) and vocal pre‐pacemaker (VPP, j–l) nuclei. Asterisks in (i) and (l) indicate neuron shown in respective insets that highlight punctate‐like label. Small, ellipsoid‐shaped ChAT positive somata are adjacent to VPN neurons (arrow in g, i). Cells adjacent to VPP show weak label (asterisk and arrow in j, l; inset in j highlights labeled cell adjacent to asterisk). The scale bar represents 50 µm in (c) for (a–c), 10 µm in (f) for (d–f), 10 µm in (i) for (g–i), 10 µm in (l) for (j–l), and 5 µm in insets in (i), (j), and (l)

In addition to serotonin and catecholamines, we investigated cholinergic input to the vocal nuclei, a third potential source of neuromodulation. Not surprisingly, neurobiotin‐labeled VMN somata and axons are positively labeled for ChAT, the enzyme that catalyzes the synthesis of acetylcholine (Figure 9a–f). By contrast, neurobiotin‐labeled VPN (Figure 9g–i) and VPP (Figure 9j–l) neurons are not co‐labeled with ChAT. Ellipsoid‐shaped cholinergic somata were, however, within and in close proximity to larger neurobiotin‐labeled VPN neurons (arrow, Figure 9g, i). At the level of VPP, there is less robust cholinergic label in neurons adjacent to similarly sized neurobiotin‐labeled neurons (asterisk and arrow, Figure 9j, l; inset, Figure 9j of cell indicated by asterisk). Cholinergic puncta within VPN and VPP (insets, Figure 9i, l) suggest cholinergic inputs to these nuclei. Due to the extensive overlap of VMN and VPN dendrites (e.g., Figure 1e, g; also see Chagnaud & Bass, 2014) and dense labeling with ChAT, we cannot distinguish cholinergic input to VMN somata or VMN and VPN dendrites.

### 3.8 Vocal CPG‐auditory hindbrain pathway

**Figure 10:**
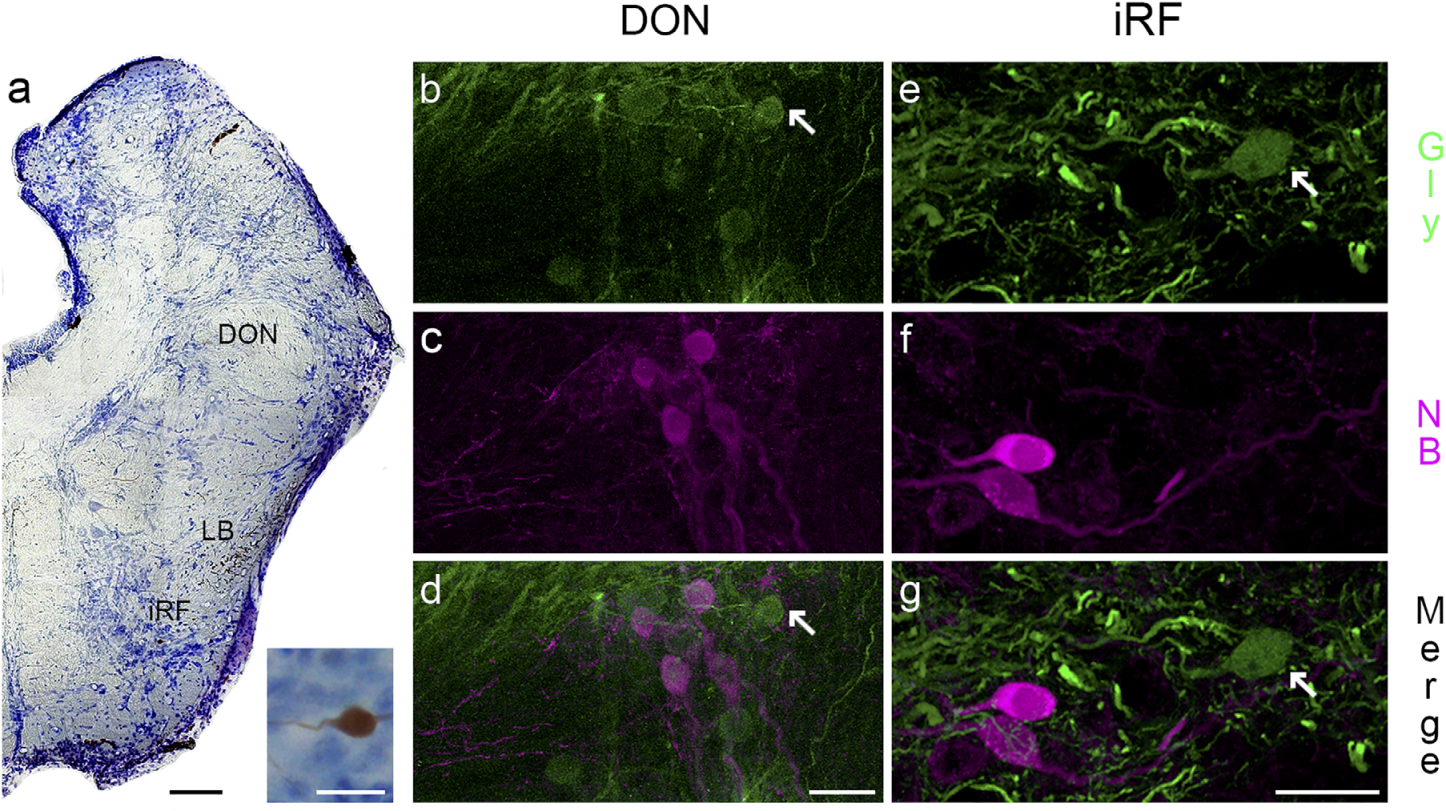
Glycinergic neurons in auditory‐recipient dorsal octovolateralis nucleus (DON), and in the inferior reticular formation (iRF) of the Gulf toadfish. Cresyl violet stained transverse section of the rostral hindbrain shows location of DON and iRF neurons (a; LB indicates lateral brainstem bundle). The inset in (a) (bottom right) shows a close‐up of a neurobiotin‐labeled iRF neuron. Glycinergic somata (green, Gly) lie close to neurobiotin‐labeled DON and iRF neurons (NB; magenta; DON: b–d; iRF: e–g). Scale bar is 50 µm in (a) and 20 µm in inset in (a), 20 µm in (d) for (b–d) and 10 µm in (g) for (e–g)

A prominent neuroanatomical and neurophysiological feature of the vocal CPG is its link to rostral hindbrain auditory nuclei, in particular a medial division of the descending octovolateralis nucleus (DON, Figure 10a) that is a part of the ascending auditory system (Bass, Bodnar, & Marchaterre, 2000; Bass et al., 1994). Although transneuronally labeled DON neurons are not co‐labeled with any of the neurotransmitters and modulators studied here, weakly labeled glycinergic neurons are immediately adjacent to transneuronally labeled DON neurons (arrows, Figure 10b–d).

Unlike prior studies in midshipman fish and toadfish (Bass et al., 1994; Chagnaud & Bass, 2014), transneuronally labeled somata also lie within the rostral hindbrain inferior reticular formation (iRF; Figure 10a shows hindbrain level and example of neurobiotin‐filled neuron in the inset). These neurons are not co‐labeled for any of the neurotransmitters and modulators studied here, although weakly labeled glycinergic neurons are nearby (arrows, Figure 10e–g). This finding is particularly intriguing because of the known involvement of the reticular formation in vocal mechanisms (e.g., Jürgens & Hage, 2007).

## 4 DISCUSSION

By combining robust transneuronal neurobiotin‐labeling of all nuclei comprising the vocal CPG of toadfish with immunohistochemical identification of inhibitory neurotransmitters and putative neuromodulators as well as a gap junction protein, we provide anatomical evidence for robust inhibitory and modulatory inputs within the vocal CPG of a teleost fish. These results complement prior neurophysiological investigations of the intrinsic and network properties of this vertebrate model for vocal‐acoustic communication. We interpret the punctate‐like label often observed as indicative of synaptic inputs, although we recognize the need to verify this assumption by combining neurobiotin‐labeling of vocal neurons with immuno‐electron microscopy. All CPG nuclei exhibit prominent GABAergic and glycinergic input. Surprisingly, and in contrast to GABAergic neurons, a subset of premotor neurobiotin‐labeled pacemaker neurons (VPN) are co‐labeled with glycine, suggesting that they are coupled to other vocal CPG neurons via gap junctions. This is consistent with extensive gap junction protein (connexin) labeling in VPN. Gap junctions are also abundant in the motor neuron population (VMN), supporting the hypothesis that gap junction coupling is prominent in the VPN–VMN circuit (Bass & Marchaterre, 1989; see also Bass et al., 1994; Chagnaud et al., 2011; Chagnaud et al., 2012). The weaker connexin label on premotor VPP somata suggests that transneuronal labeling of VPP is due to gap junction coupling between VPP axons and the somata and/or dendrites of VMN and/or VPN neurons. As discussed below, the evidence for catecholaminergic, serotonergic, and cholinergic label suggests a role for these neuromodulators along with inhibitory neurotransmitters and gap junctions in establishing the rhythmic, oscillatory‐like output of the vocal CPG that is translated directly into the temporal features of vocal behavior.

### 4.1 GABAergic and glycinergic neurons within the vocal CPG

#### 4.1.1 GABA

In line with a previous study of midshipman fish that used a GABA antibody shown to be specific in oyster toadfish*, Opsanus tau* (Holstein et al., 2004), GABAergic neurons are positioned lateral to VMN in the Gulf toadfish. These neurons are the likely source of the strong GABAergic input to VMN (Chagnaud et al., 2012). GABAergic neurons are also found within VPN and adjacent to VPP. None of the GABAergic neurons are co‐labeled with neurobiotin, suggesting a lack of gap junction coupling to other vocal CPG neurons. Neurophysiological studies in midshipman fish are consistent with these findings. Injection of bicuculline, a competitive GABA_A_ receptor antagonist, into VPP leads to an increase in call duration, the vocal parameter coded by VPP (Chagnaud et al., 2011). Intracellular recordings of VMN neurons together with local bicuculline injections into VMN show that GABAergic action at first distorts and then eliminates VMN activity, revealing that GABAergic inhibition is essential to generate vocal signals (Chagnaud et al., 2012).

Activation of GABAergic neurons that inhibit VMN might originate from within or outside of the vocal CPG. VPP is a well‐suited candidate from within the vocal CPG, as VPP neurons fire just before and for the duration of the vocal behavior (Chagnaud et al., 2011). A candidate from outside of the vocal CPG would be the midbrain periaqueductal gray (PAG). The PAG activates the vocal CPG via direct input to the duration coding VPP neurons (Chagnaud et al., 2011; Goodson & Bass, 2002; Kittelberger, Land, & Bass, 2006) and itself may influence call duration (Kittelberger et al., 2006). Axon collaterals from the PAG might activate GABAergic neurons adjacent to VMN and VPN to prime the vocal system and to sculpt vocal activity. If GABAergic neurons tonically inhibit VMN during vocal behavior, this prolonged GABAergic action would facilitate a de‐inactivation of voltage dependent sodium channels of the weakly excitable VMN motor neurons (Chagnaud et al., 2012).

#### 4.1.2 Glycine

Glycinergic neurons are found at all levels of the vocal CPG. Together with GABA, there are three types of inhibitory neurons in VPN: those labeled only for either glycine or GABA, and glycinergic neurons co‐labeled with neurobiotin. All three types may provide input to VMN and/or other VPN neurons that are only labeled with neurobiotin. The glycine‐neurobiotin population is especially interesting as the co‐label suggests that these neurons are coupled electrically via gap junctions to other vocal CPG neurons; in support, there is strong Cx 35/36 label in VPN. Due to extensive gap junction coupling in the vocal network, the location of the electrotonic coupling (dendro‐dendritic, dendro‐axonic, axo‐axonic) of neurobiotin‐glycine neurons to VMN and/or other VPN neurons remains elusive.

There are three possible, non‐exclusive functions for the neurobiotin‐glycine co‐labeled VPN neurons. The first is that VPP's direct activation of VPN neurobiotin‐only neurons would co‐activate neurobiotin‐glycinergic neurons via electrotonic coupling. This co‐activation could, in turn, inhibit non‐glycinergic VPN neurons via a chemical (glycinergic) synapse. This form of recurrent inhibition could lead to the oscillatory‐like firing pattern that characterizes VPN neurons during vocal activity (Bass & Baker, 1990; Chagnaud et al., 2011). The oscillatory properties of VPN neurons would then be a network property of the neurobiotin‐only labeled VPN population rather than an intrinsic property of these neurons. Electrophysiological recordings from VPN in midshipman fish strengthen this hypothesis as current injection into VPN neurons does not induce the characteristic firing frequency displayed during vocal activity (Chagnaud et al., 2012).

Second, gap junction coupling between VMN motor neurons and neurobiotin glycine co‐labeled VPN neurons could lead to a direct modulation of motor neuron activity by (VPN) premotor neurons. VMN activity could depolarize neurobiotin‐glycine co‐labeled VPN neurons via electrical synapses and thereby activate them. The activation of these VPN neurons could, in turn, modify the firing pattern of neurobiotin‐only labeled VPN neurons via a chemical glycinergic synapse. As described above, this would lead to a change in the firing frequency of VPN neurons and thus modify VMN activity. Similar motor–premotor coupling has recently been shown in zebrafish and *Drosophila* locomotor networks in regard to motor rhythm control (Matsunaga, Kohsaka, & Nose, 2017), as well as in the *Xenopus* vocal pattern generator (Lawton, Perry, Yamaguchi, & Zornik, 2017).

A third possible function of the glycine‐neurobiotin co‐labeled VPN neurons is that they contribute to the synchronous firing of the VMN neurons during vocal behavior (Chagnaud et al., 2012). Depolarization of the neurobiotin‐glycine co‐labeled neurons in VPN via electrical coupling could inhibit VMN neurons via chemical glycinergic synapses. This would result in the suppression of VMN activity directly after VMN neurons are activated, thus leading to a precise and rapid suppression of VMN activity and repolarization of its motor neurons. This VMN activity dependent inhibition would foster synchronized firing of vocal motor neurons by preventing sustained action potential firing. The coupling of glycinergic neurons to the VPN, VMN or both nuclei, could thus contribute to the extreme synchrony and temporal precision that characterizes the VPN–VMN circuit in toadfishes (Chagnaud et al., 2011; Chagnaud et al., 2012). Glycinergic innervation of hindbrain motor neurons involved in vocal production has also been shown in zebra finches, *Taeniopygia guttata* (Sturdy, Wild, & Mooney, 2003). Whether a subset of these glycinergic neurons are also gap junction coupled to vocal motor neurons, as in toadfish, remains unknown.

### 4.2 Neuromodulators in the vocal CPG

#### 4.2.1 Serotonin

In agreement with reports for midshipman fish and the oyster toadfish that used different antibodies from the one used here (Forlano, Nielsen, & Timothy, 2011; Marchaterre, Baker, Baker, & Bass, 1989; Timothy, Ghahramani, Gorbonosov, Ferrari, & Forlano, 2015), we report serotonergic input throughout the vocal CPG. Serotonergic neurons within the VMN clearly provide one source of serotonergic innervation to the VMN. Potentially, these serotonergic neurons could also innervate adjacent VPN neurons and the more rostral VPP population; the dense serotonergic input to VPN, in particular, makes it impossible to assess this here. Another potential source of serotonergic input to the vocal CPG are serotonergic neurons within the inferior raphe nucleus that have projections throughout the brainstem of fish (e.g., Corio, Peute, and Steinbusch, 1991; Grant, Clausse, Libouban, and Szabo, 1989; Kah & Chambolle, 1983).

Serotonergic input to all three vocal CPG nuclei suggests that serotonin can modulate one or more of the three main vocal parameters—amplitude, frequency and duration, all of which remains to be tested using electrophysiology and behavioral assays. Serotonin is known to initiate vocal motor patterns in an isolated brain preparation of *Xenopus laevis* (Rhodes, Yu, & Yamaguchi, 2007). In contrast, systemically administered serotonin agonists terminate territorial calling in the Puerto Rican coquí frog, *Eleutherodactylus coqui* (Ten Eyck, 2008). While the influence of serotonin on vocalization could be species‐dependent, these contrasting effects of serotonin in frogs might reflect methodological differences using bath application of serotonin in an isolated brain preparation versus a systemic injection in intact animals.

Serotonergic projections to vocal populations have also been observed for cat respiratory motor neurons involved in controlling airflow for vocalization (Holtman, 1988) as well as to the nucleus of the arcopallium in zebra finch (Wood et al., 2011). Similarly, *X. laevis* laryngeal pre‐motor and motor neurons receive serotonergic input (Yu & Yamaguchi, 2010). Thus, serotoninergic input to vocal motor areas appears to be a conserved pattern among vocal vertebrates.

#### 4.2.2 Catecholamines

In accordance with previous studies in midshipman fish using different antibodies (Forlano et al., 2014; Goebrecht et al., 2014), we show dense catecholaminergic input to the vocal CPG in Gulf toadfish. Like midshipman fish (Forlano et al., 2014), neurons located dorsolateral to the VMN and within the area postrema likely provide catecholaminergic input to VMN neurons in Gulf toadfish. Additional catecholaminergic inputs from other neurons within the central nervous system, for example, spinal projecting dopaminergic neurons in the diencephalon as suggested for midshipman fish (Forlano et al., 2014), can, however, not be excluded.

Catecholamines might alter vocal production in toadfishes at forebrain levels such as the anterior hypothalamus (Forlano & Bass, 2011; Forlano et al., 2014; Kittelberger et al., 2006) as well as at the level of the hindbrain vocal CPG and midbrain (Heisler & Kittelberger, 2012). Consistent with the anatomical evidence for catecholaminergic input to VMN, dopamine receptor subtypes are upregulated in midshipman VMN compared to the surrounding hindbrain (Feng, Fergus, & Bass, 2015). Catecholamine innervation of VMN is also denser in sneaker type II male compared to highly vocal, advertisement calling type I male midshipman fish, suggesting an inhibitory role of catecholamines (Ghahramani et al., 2015; Goebrecht et al., 2014; for behavior see also Brantley & Bass, 1994). A preliminary report shows that injection of dopamine into the PAG of midshipman suppresses vocal motor output, while dopamine receptor antagonists partially block this inhibition (Heisler & Kittelberger, 2012). Similarly, dopamine‐like receptor activity negatively influences advertisement calling in green tree frogs, *Hyla cinerea*, after intraperitoneal injection of dopamine agonists (Creighton et al., 2013). Noradrenaline reduces activity in a brain slice preparation of the robust nucleus of the arcopallium, the premotor song control nucleus in songbirds, after bath application (Solis & Perkel, 2006). Perhaps contrary to this apparent inhibitory role for catecholamines, catecholaminergic innervation of vocal areas is more abundant in singing male canaries, *Serinus canaria*, compared to nonsinging females (Appeltants et al., 2003).

#### 4.2.3 Acetylcholine

The pattern of ChAT label observed for the Gulf toadfish matches that previously shown for the VMN of midshipman fish using a different antibody (Brantley & Bass, 1988). Like serotonin and catecholamines, there is cholinergic input to VPN and VPP. While injections of cholinergic antagonists into vocal midbrain sites of squirrel monkeys, *Saimiri sciureus* show no effect on vocal production (Jürgens & Lu, 1993), in vivo and in vitro application of acetylcholine and its agonists to respiratory pacemaker‐like neurons of the pre‐Bötzinger complex show increased duration, frequency and amplitude of spontaneous inspiratory bursts (Burton, Johnson, & Kazemi, 1997; Monteau, Morin, & Hilaire, 1990; Murakoshi, Suzue, & Tamai, 1985; Shao & Feldman, 2000). Perhaps acetylcholine plays a more widespread role in enhancing premotor activity in hindbrain pattern generators, including the vocal CPG of toadfish.

### 4.3 Vocal CPG projections to brainstem areas

Like previous studies in midshipman fish and Gulf toadfish (Bass et al., 1994; Chagnaud & Bass, 2014; Weeg, Land, & Bass, 2005), the vocal CPG is connected to auditory hindbrain neurons with evidence for nearby glycinergic neurons. Although previous electrophysiological experiments reveal that inhibition is important in determining vocal CPG output (earlier section), it is also known to play an important role in brainstem mechanisms of vocal‐acoustic integration in mammals (Smotherman, 2007). Unexpectedly, neurobiotin‐labeled cells are also located in the iRF in close proximity to glycinergic neurons. After carefully revisiting the material used by Chagnaud and Bass (2014), neurobiotin‐labeled iRF neurons are also observed in that material, although the iRF labeling in that study was quite weak compared to the robust labeling that we observe here and so was easily missed. GABAergic neurons are also found in the iRF of the African cichlid fish, *Astatotilapia burtoni*, that is also sonic (Maruska, Butler, Field, & Porter, 2017). Studies of squirrel monkeys demonstrate the reticular formation's involvement in vocal production (Jürgens & Hage, 2007). The PAG and other vocal midbrain sites in midshipman are also connected to the reticular formation (Goodson & Bass, 2002; Kittelberger & Bass, 2013). While suggestive, the involvement of the reticular formation in vocal patterning or production in toadfishes awaits neurophysiological investigation.

## 5 CONCLUDING REMARKS

In this study, we show prominent inhibitory and neuromodulatory input at all levels of the toadfish vocal CPG that suggest a suite of neurophysiological mechanisms to achieve a variety of motor programs (Gjorgjieva, Drion, & Marder, 2016; Harris‐Warrick & Marder, 1991), with a single set of topographically separate nuclei—VMN, VPN, VPP—resulting in context‐dependent vocal signals. The presence of inhibitory transmitters such as GABA and glycine, along with gap junction coupling within each of the CPG nuclei, likely contribute to determining two predominant features of the vocal CPG—extreme temporal precision and synchrony (Chagnaud et al., 2012). How serotonin, catecholamines and acetylcholine interact with these transmitters to shape vocal production remains to be shown. The proposed evolutionarily conserved organization of vocal CPGs (Bass et al., 2008; Bass et al., 1994), together with the available neurophysiological evidence in toadfishes and other sonic species of vertebrates, suggests that comparable neurochemically dependent mechanisms are present in other vertebrate vocal CPGs.

## CONFLICT OF INTEREST

The authors declare that there are no conflicts of interest.

## Supporting information

Additional Supporting Information may be found online in the supporting information tab for this article.

Supporting Figure S1
