# Supplementary figures and images for: Inhibitory and modulatory inputs to the vocal central pattern generator of a teleost fish
Source: J Comp Neurol. 2018 Feb 28;526(8):1368–88. doi: 10.1002/cne.24411 (PMC5901028; doi:10.1002/cne.24411)

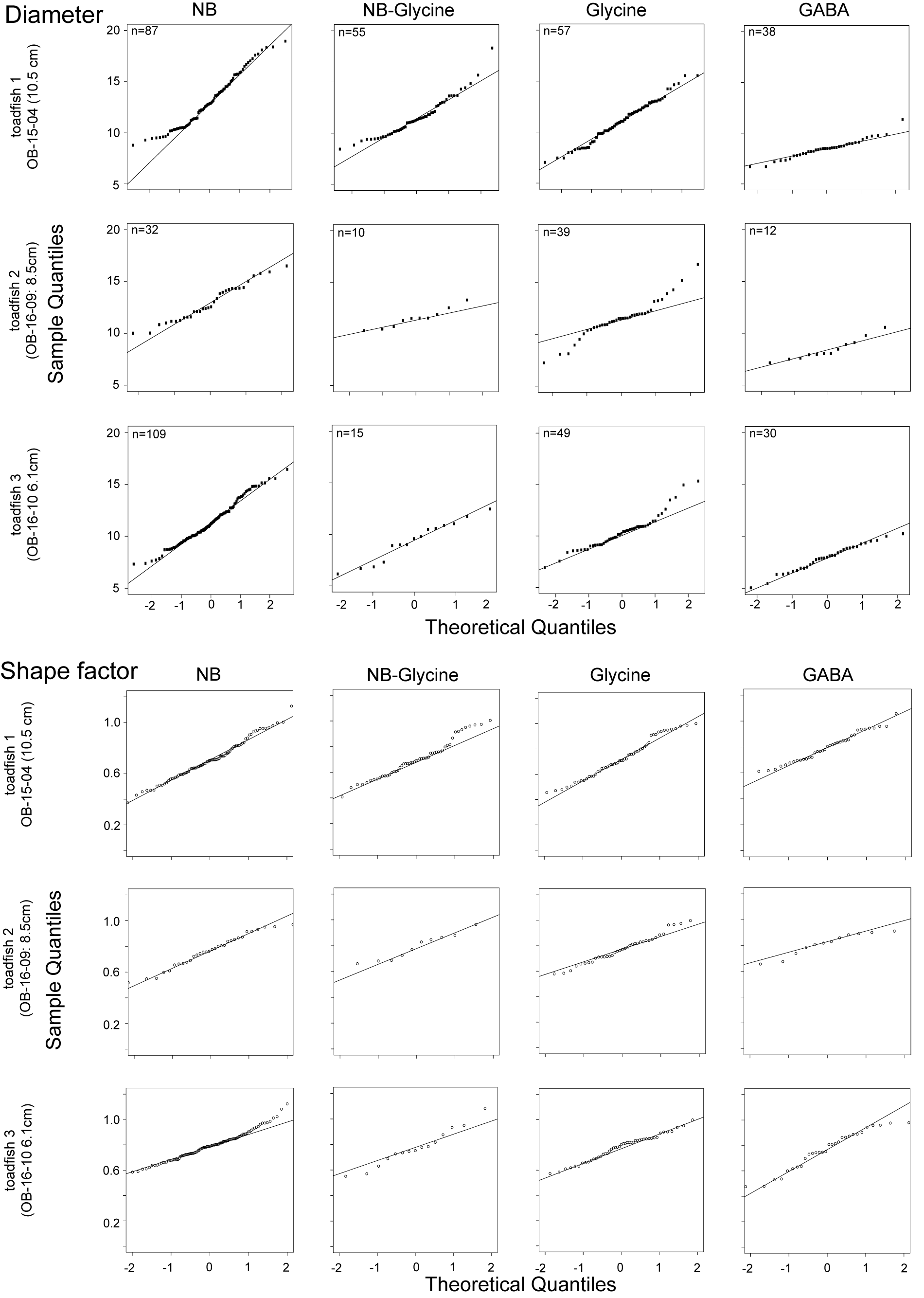

Supplement: Supplementary file 1 — Supporting Figure S1 [file CNE-526-1368-s001.tif]
